# Stakeholders’ experiences in implementation of rapid changes to the South African prevention of mother-to-child transmission programme

**DOI:** 10.4102/phcfm.v10i1.1788

**Published:** 2018-11-15

**Authors:** Hlolisile W. Chiya, Joanne R. Naidoo, Busisiwe P. Ncama

**Affiliations:** 1 School of Nursing and Public Health, University of KwaZulu-Natal, South Africa; 2 Department of Nursing Science, Nelson Mandela University, South Africa

## Abstract

**Background:**

South Africa’s prevention of mother-to-child transmission (PMTCT) of the human immunodeficiency virus (HIV) programme has undergone rapid changes in the last two decades. Initially, the provision of single antiretroviral therapy was based on eligibility criteria in the year 2001, which later changed to combination therapy. This was aimed at preventing mother-to-child transmission of HIV. Since 2015, all pregnant women were eligible for antiretroviral treatment regardless of their CD4 count. Although significant strides were made to reduce mother-to-child transmission of HIV, increased efforts are required to meet UNAIDS targets, World Health Organization (WHO) elimination framework goals and sustainable development goals to eliminate new HIV infections in children and ending the HIV epidemic by 2030.

**Aim:**

The aim of the study was to explore healthcare workers’ experiences and patient perceptions of the implementation of rapid changes to the PMTCT programme in four public healthcare facilities.

**Setting:**

The study was conducted in the four public healthcare facilities within the two highly HIV-burdened districts of iLembe and eThekwini in KwaZulu-Natal province, South Africa.

**Methods:**

This study used a qualitative, exploratory, descriptive study design using interviews and focus group discussions. Participants were selected using purposive sampling. Following verbatim transcription of the data, thematic data analysis was used through data reduction and data display and the emergence of four themes.

**Results:**

A total of 61 stakeholders were interviewed. Four major themes emerged: (1) impact of poor health system design, (2) impact of poor communication of changes, (3) contextual factors affecting innovation in healthcare and (4) skill deficit in change management and forward planning.

**Conclusion:**

A healthcare system more responsive to the experiences of healthcare workers and pregnant women is required to effectively implement changes in priority programmes.

## Background

The prevention of mother-to-child transmission (PMTCT) of human immunodeficiency virus (HIV) programme is a public health intervention to reduce transmission of HIV from mother to child. The programme provides antiretroviral treatment (ART) to HIV-positive pregnant women to stop them from transmitting the virus to their infants.^1^ Without treatment, the likelihood of HIV passing from mother to child is 15% – 45%.^1,2,3,4^ However, ART and PMTCT cascade included interventions among others: antenatal services, HIV testing during pregnancy, use of ART by pregnant women living with HIV, safe childbirth practices and appropriate infant feeding, uptake of infant HIV testing and other postnatal healthcare services can reduce the risk to below 5%.^5^

Globally, PMTCT programmes have faced rapid changes because of HIV evolving and in response to the World Health Organization (WHO) recommendations.^2,6^ Since 2001, the WHO has revised guidelines to the PMTCT programme more than five times, and the latest change to the programme was the 2016 revised guidelines recommending the provision of lifelong ART to all children, adolescents and adults, including all pregnant and breastfeeding women living with HIV, regardless of CD4 cell count.^1,3,4,7,8^ In 2011, UNAIDS launched the Global Plan aimed at prioritising a set of countries that, in 2009, accounted for 90% of the global total of pregnant women living with HIV who were in need of services to prevent mother-to-child transmission of HIV. The plan targeted goals, amongst others, to reduce new HIV infections in children by about 90% by 2015 and increase the number of pregnant women with HIV receiving ART to 90% by 2015.^2,6,9^ The WHO identified 22 priority countries, including South Africa, to implement the plan. It was hoped that if the priority countries scale up interventions effectively, more than 250 000 new HIV infections annually will be prevented in children. Disparities were observed in the countries’ response to achieving the targets.^6^

The key processes for the HIV programme, according to the WHO,^1,3^ included four pillars:

- Preventing new HIV infections amongst women of childbearing age.
- Preventing unintended pregnancies amongst women living with HIV.
- Preventing HIV transmission from a woman living with HIV to her baby.
- Providing appropriate treatment, care and support to mothers living with HIV and their children and families.

By 2016, South Africa was one of the six countries to have shown significant reduction in new HIV infections amongst children. The country progressed from 73% reduction in new HIV infections in children in 2013 to about 84% reduction in 2015.^6^ The percentage of pregnant women living with HIV receiving ART increased significantly beyond the global target of 90%.

Countries are expected to accelerate their efforts on the progress made to close the remaining gaps in working towards the goal of ending the HIV epidemic by 2030. This is in line with UNAIDS targets, the WHO elimination framework and the sustainable development goals seen as a platform for countries to utilise in eliminating new HIV infections in children.^10^

In addition, South Africa’s National Strategic Plan 2012–2016^11^ outlines how the country will respond to the prevention and treatment of HIV and AIDS, tuberculosis and sexually transmitted infections over the next five years.

In the context of South Africa, the PMTCT programme has experienced several policy changes^12,13,14,15,16^ as demonstrated (See Tables 1 and 2^15^), aligning with the recommendations by the WHO.^4^ The PMTCT programme aims to reach out to all women before and during pregnancy, through labour and delivery, and through the postnatal period up to 18 months.^17,18^

**TABLE 1:** Demographic details of participants.

Variable	Health workers	Community caregivers	Pregnant women	%	Total
**Age group**					
18–24	3	0	21	39	24
25–31	6	0	9	25	15
32–38	7	1	8	26	16
39–45	3	1	2	10	6
Total	19	2	40	100	61
**Marital status**					
Single	11	1	24	59	36
Married	7	1	16	39	24
Widowed	1	0	0	2	1
Total	19	2	40	100	61
**Educational status**					
No education	0	0	0	-	0
Primary education	0	0	7	11	7
Secondary education	0	0	17	28	17
Higher education	4	2	12	30	18
Diploma or degree	15	0	4	31	19
Total	19	2	40	100	61

**TABLE 2:** Evolution of the programme for prevention of mother-to-child transmission in South Africa.

Year	PMTCT programme interventions
1998–1999	PMTCT programme started in two sites in Khayelitsha, Western Cape despite national policy.
2000	13th International HIV Conference in Durban, KZN. Data were presented which highlighted the effectiveness of antiretroviral regimen to reduce mother-to-child transmission of HIV.
2001	Two research sites established by NDOH in each province for 2-year period as pilot sites, to understand the operational challenges of introducing antiretroviral therapy in pregnancy for PMTCT.
2001	Court challenge to pilot programme lead by TAC, representing civil society. December 2001: SA government ordered by the court to develop an effective national programme to reduce MTCT by 2002.
2002	Government unsuccessful in appealing the court decision, as a result of which the PMTCT programme commences.
2003	New operational plan introduced for treating HIV-positive people. Nevirapine programme introduced; treatment extended to all pregnant women and children infected with HIV, with related healthcare services such as voluntary counselling and testing.
2004	Comprehensive care management and treatment of HIV-infected individuals. Pregnant women with CD4 count less than 200 cells/mm^3^ eligible for highly active antiretroviral treatment (HAART).
2008	DOH update of PMTCT policy to include dual therapy with azidothymidine and NVP from 28 weeks’ gestation, nevirapine treatment for pregnant women during labour and for their babies within 72 h of delivery, and HAART for pregnant women with CD4 cell count less than 200 cells/mm^3^.
2008	Launch of the national PMTCT accelerated plan (9A-Plan) aimed at reduction of MTCT from 12% in 2008 to less than 5% by 2011 in line with National Strategic Plan (2007–2011).
2009	President Zuma’s speech on World AIDS Day (1 December each year) outlines changes to be implemented in 2010 to demonstrate political leadership in the fight against HIV.
2010	DOH revises the PMTCT policy to include lifelong HAART for HIV-positive women with CD4 count less than 350 cells/mm^3^ and dual ART from 14 weeks onwards for pregnant women with CD4 above 350 cells/mm^3^ (Option A of WHO); all infants to take NVP daily for 6 weeks and to continue for all breastfeeding infants whose mothers were on HAART, for postnatal transmission reduction.
2011	Phasing out of free formula following a national conference on breastfeeding; Minister of Health endorses a breastfeeding policy where breastfeeding to be used exclusively at public health facilities, with formula milk reserved for medical indications.
2011	In line with global agencies, DOH develops a national action framework for eliminating MTCT of HIV.
2013	Revised guidelines to include fixed-dose combination (FDC), Option B, according to new WHO recommendations; the routine offers of ART irrespective of CD4 count or clinical stage will improve treatment access; simplified approaches, including a common regimen of tenofovir, lamivudine or emtricitabine and efavirenz as a FDC tablet for women and older children living with HIV, should also improve uptake.
2015	Revised ART guidelines. Government announces (in budget speech by Dr Motsoaledion in July 2014) that South Africa will adopt an Option B+ as per WHO recommendations; this allows all HIV-positive pregnant women to be started on ART for lifelong therapy regardless of their CD4 count; women who are breastfeeding and women who are within 1 year post-partum to be initiated to eliminate MTCT; guidelines effective from 1 January 2015.
2016 Sept 1	Universal testing and treating implemented. All HIV-tested positive population regardless of pregnancy, or post-partum, or their CD4 count are eligible to be initiated on lifelong ART.

*Source*: Barron P, Pilly Y, Doherty T, et al. Eliminating mother-to-child transmission in South Africa. https://doi.org/10.2471/BLT.12.106807

KZN, KwaZulu-Natal; ART, antiretroviral treatment; PMTCT, prevention of mother-to-child transmission of HIV; MTCT, mother-to-child transmission of HIV; FDC, fixed-dose combination; WHO, World Health Organization; NDOH, National Department of Health; DOH, Department of Health; NVP, nevirapine; TAC, Treatment Action Campaign.

South Africa has made considerable progress towards the elimination of new HIV infections in children; however, with constant changes brought about by the evolving nature of the HIV epidemic (See Tables 1 and 2), it remains crucial that the challenges of implementing rapid changes to the PMTCT programme are identified and addressed. Hence, there is a need to understand the experiences of healthcare workers, as service providers, who are involved in implemention as well as consumers who are on the receiving end.

### Aim of the study

The aim of this study was to explore healthcare workers’ experiences and patient’s perceptions of the implementation of changes to the PMTCT programme.

### Study period

The study was undertaken between 2013 and 2016 when the national consolidated guidelines for the PMTCT and the management of HIV in children, adolescents and adults were still in use in South Africa.^17^

### Contributions made by the study

The study findings provide an understanding of the implications of the rapidly changing PMTCT programme implementation as experienced by healthcare workers and consumers of the programme in public health care settings. This is important in a country working towards the goal of eliminating new HIV infections in children.

## Research methods

### Study design

The study adopted a qualitative approach using exploratory descriptive design in seeking to understand perceptions of health care workers and patients during implementation of rapid changes in the PMTCT programme in the selected health care facilities. According to Denzin and Lincoln,^19^ qualitative researchers attempt to interpret or make sense of a phenomenon in its natural setting and to discover the meaning people bring to the phenomenon under study.

This study was conducted between 2013 and 2016, in the four selected public healthcare facilities of the iLembe and eThekwini health districts in KwaZulu-Natal (KZN), South Africa. One of nine provinces in South Africa, KZN has the highest population at more than 10 million (StatsSA 2016).^19^ Within KZN, there are 11 health districts, which form the next level of government below provincial level. The KZN provincial HIV prevalence saw an increase from 37.4% in 2011 and 2012 to 40.1% in 2013, which was a moderate increase of 2.7%.^18^ The two districts under study recorded the highest HIV prevalence in pregnancy of above 40%, which is amongst the highest in the country (iLembe = 45.9%, eThekwini = 41.1%). According to the WHO,^1^ this exposes children to risk of transmitted HIV. The two study districts were selected using the District Health Information System,^21^ which has records of all the health facilities for KZN with their disease burden.

Permission was sought prior to research engagement from the Provincial Directorate for Health in KZN as well as from the respective public healthcare facilities, healthcare workers and from individual participants. Detailed information was given and written informed consent was sought prior to participation, including consent for the interviews to be recorded on audio tapes. Access to the study information was limited to the researcher and the study supervisors. Data were stored in a password-protected laptop and all written information was stored in a locked cupboard.

### Trustworthiness

Qualitative studies follow a naturalistic interpretive paradigm based on the philosophical belief in multiple perspectives. According to Glaser and Strauss,^22^ trustworthiness is the extent to which one believes in the research findings. To enhance trustworthiness, the criteria for credibility, transferability, dependability and conformability were applied. Credibility was ensured through use of self-administered questionnaires, focus group discussions (FGDs) and in-depth interviews. Use of multiple case study settings and various data sources ensured triangulation of data collected and study findings. Using audio tapes, interview transcripts and field notes to capture the information allowed the researcher to ensure that the information was not distorted and was a true reflection of the information collected. Through the consciousness of the researcher’s personal attitude, opinions, experience and expectations as a healthcare worker who was once employed for the PMTCT programme, she endeavoured to remove bias.^23^

Transferability was achieved through studying four health facilities in their context using similar data collection methods.^24^ Recordings of the interviews enhanced the authenticity and were supplemented by inclusion of verbatim quotes from research participants to describe findings, which assisted in achieving transferability.

Dependability ensured consistency and the ability for other researchers to replicate the study.^25^ This was achieved through the provision of the details on study design, method used for data analysis and data collection methods. To ensure conformability, collected information was verified with key informants and study participants throughout the process of data collection.

### Population and sampling

The target population consisted of various categories of nursing staff including community caregivers as well as patients in the public healthcare facilities (hospitals and primary healthcare facilities) in the iLembe and eThekwini districts in KZN. Non-probability purposive sampling of participants and study sites was used to identify participants who were knowledgeable and understood the programme for PMTCT to share their experiences during rapid changes to the PMTCT programme.^26^ Also, sampling was based on working in the area where the programme was implemented based on eligibility criteria for the study. This enabled the researcher to select participants who could answer the question of interest.^25^ The inclusion criteria included healthcare workers and community caregivers with more than 1 year’s experience working in a unit providing the maternal and child health programme, including the PMTCT programme, in the four selected public healthcare facilities in the study districts. The study sites were selected based on high HIV prevalence in pregnant women which was over 40%.^18^

The sampling frame was the list of public healthcare workers, including community caregivers, employed in the four selected sites. The sampling unit was any healthcare worker or community caregiver who had worked for over a year with the PMTCT programme in the four selected study sites. Patients were purposively sampled in their respective four public healthcare facilities who met the criteria of being pregnant, attending antenatal care services and receiving PMTCT services in the four study sites. The sampling frame included a list of all pregnant women attending antenatal care in the four study sites. The sampling unit was any pregnant women attending antenatal care who had been receiving PMTCT services in the selected sites. The sample consisted of 19 nursing staff, 2 community caregivers and 40 pregnant women who were purposively selected for this study.

### Data collection

In this study, the data collection setting was the four selected public healthcare facilities in the selected health districts. Data collection occurred during the lunch break and in the late afternoons to prevent disruptions of the activities within the health facility. In this study, the data collection sought to analyse the perceived experiences regarding the implementation of changes to PMTCT by key stakeholders.

### Data collection methods

Data collection tools were developed in consultation with the programme manager for the PMTCT programme who is considered an expert in this programme for KZN. The purpose was to gain an understanding of the PMTCT programme within the contexts. Data collection occurred in two forms, namely for the health workers as well as for the patients accessing the PMTCT services. Two sets of data collection methods were used to understand the experiences and perceptions of various stakeholders regarding the implementation of changes to PMTCT in their setting. These measures ensured face validity and construct validity. A semi-structured interview guide was developed for patients to assess their perceptions on the changes to the programme for PMTCT in their setting. This form of data collection enabled face-to-face conversation using an interview guide to remind the researcher of issues, topics and key concepts to cover, but was flexible as it allowed the researcher to explore issues that needed more clarity.^25^ The aim of interviews was to explore patients’ perceptions of the changes for the PMTCT programme. For the healthcare workers, an FGD interview guide was developed as a data collection tool to allow for collecting a diversity of meaning, opinions, experiences and differences that healthcare workers provide in each context. The FGDs were recorded on audio tapes following permission to record from participants.

Eligibility criteria included pregnant women who had been attending the selected study site and were receiving PMTCT services. Interviews with key informants were conducted after completion of routine visits by consenting pregnant women. The objective was to describe the perceptions and understanding of pregnant women as consumers of the implications of rapid changes to the PMTCT programme. A summary of data collection is provided in Table 3.

**TABLE 3:** Data collection summary.

Participants and/or key informants	Method of data collection	Sampling criteria	Number sampled (*N* = 61)
Healthcare workers incl. CCGs	Focus group discussions	Purposive sampling	21
Pregnant women	Semi-structured in-depth interviews	Purposive sampling	40

CCGs, community care givers, which in this study refers to the care givers that work in defined communities to provide households with basic health needs.

### Data analysis

A qualitative approach using multiple sources of evidence to collect data offered a comprehensive understanding of concepts and meaning as perceived by multiple stakeholders on the implementation of PMTCT programme changes.^19^ Verbatim transcription of all conducted interviews and recorded data from FGDs was performed manually and was accompanied by checking of transcripts with participants to seek clarity and ensure quality of information collected. An exploratory approach was adopted using thematic content analysis. The use of thick description^26^ during analysis together with quotes from interviews and FGD enabled understanding of participants’ perceptions and their experiences in the implementation of the rapid changes to the PMTCT programme.

Data were collected from interviews and FGD transcripts as described by Braun and Clarke.^21^ The aim was to gain insight into the experiences and perceptions of stakeholders in the implementation of changes through an inductive process. This inductive process allowed for building patterns, categories and themes from the bottom up through organisation of data into more abstract units of information, driven by the research question, for presentation of a rich description of stakeholders’ experiences and perceptions. The codes were grouped manually into similar concepts and constructs that reflected stakeholder experiences and themes identified after data collection, as emerging grounded in the original data until saturation was reached. Unique identifiers were allocated to participant quotes for identification of site codes (S1 to S4), as well as staff and patient (patients or health care workers) categories.

## Results

### Demographic details

A total of *n* = 21 health workers and *n* = 40 patients participated, making a total of 61 participants (Table 1). The majority of patients (53%) were between the ages of 18 and 24 years, with 38% of health care workers of the same age category. In addition, 60% of patients reported to be single, with almost the same proportion (57%) of healthcare workers also being single. Healthcare workers’ highest educational qualification was either diploma or degree, and 43% patients reported secondary education as the highest educational qualification. Table 1 summarises demographic data, whilst Table 4 describes the experience of healthcare workers who were considered experts in the study.

**TABLE 4:** The experience of health workers who were interviewed during focus group discussion.

Variables (*N* = 19)	Total number
Advanced HIV and/or AIDS management training	1
Advanced midwifery	3
More than 10 years’ experience	7
Less than 10 years’ experience but more than 5 years	8
Degree and/or diploma	15

## Qualitative data analysis

### Key themes emerging from analysis of transcripts

Four key themes emerged from the analysis: (1) impact of poor health system design across facilities, (2) impact of poor communication of changes, (3) contextual factors affecting innovation in healthcare facilities and (4) skills deficits in change management and forward planning.

### Impact of poor health system design

There appears to be differences across various healthcare facilities in the implementation of changes in the PMTCT programme.

It emerged that the support system at the implementation level plays a pivotal role in providing direction and leadership for change adoption as health workers realised that changes had been continuous:

‘… as much as there are frequent changes with us it becomes easy because our manager supports us after we receive workshop.’ (S2, P5, healthcare worker, 27 years, female)

This theme in this context was used to include how things influence one another within the ecosystem, in relation to, for example, leadership engagement, and buy-in from the facility manager and staff to lead change and guide implementation:

‘… she gathers us, to see if we all know what is expected of us so when we start doing it we are certain what we are doing.’ (S2, P11, healthcare worker, 36 years, female)

‘I have seen the programme changing from one pill to three pills given to women … now one combination pill.’ (S1, P8, healthcare worker, 41 years, female)

‘… changes of eligibility of programme had been changing a lot from giving after 28 weeks to now giving when tested positive.’ (S3, P2, healthcare worker, 31 years, female)

It emerged that initial confusion in understanding of the change was felt for a time by the clinicians as well as patients during initial stages of implementation of the changes. See quotes from a patient and clinician below:

‘… us (patients) … it takes time for us to know about these things … and I always read papers and I heard news that we will get one pill … that is good but until you get to the clinic it becomes not clear.’ (S2, P7, pregnant woman, 30 years, female)

‘I will read and read to understand the change, what the difference from the old guideline so I can understand this one, but that takes time and it never easy.’ (S2, P9, healthcare worker), 36 years, female

Frequent auditing by the manager seemed to be one system that assisted with monitoring in the setting. As stated by a healthcare worker:

‘… she conducts audits that will ensure that we understand, and if she sees that there are gaps, she do not hesitate to call a meeting and brief us.’ (S1, P8, healthcare worker, 41 years, female)

This included engagement of a community caregiver (CCG) in the session to update staff, which was highlighted as important:

‘… she will then share some ideas of how she thinks we need to plan and implement, and this is communicated to everyone including our CCGs.’ (S1, P18, healthcare worker, 39 years, female)

It became apparent that planning by a manager prior to implementing changes was seen by others as vital to buying into the change, as it prepares everyone for what is to come, as per the following statements:

‘… our manager draws up a plan on how we will do in our facility and she shares that during meetings.’ (S4, P17, healthcare worker, 42 years, female)

‘… they try but sometimes we get this information very late and we wonder why so late … but we happy that we have few pills now.’ (S2, P10, pregnant woman, 38 years, female)

The support by the CCG was highlighted as crucial by pregnant women to help ask questions that may have not been provided in the health facility:

‘… this all become very confusing, until time when they (CCG) visit me, she asks me whether I understand and know, and I like that because I can ask her (CCG) anything.’ (S4, P5, pregnant woman, 21 years, female)

Whilst the managers at some places seem to be actively involved and played a pivotal role in supporting the implementation, there were some concerns about managers who were less involved, leaving it to other participants to justify changes and provide reasons; hence, it was clear that the disengagement of the manager had a negative affect:

‘… our manager is sometimes not that much involved because we are the ones to tell her what is new.’ (S2, P13, healthcare worker, 33 years, female)

The lack of involvement by the managers is a bottleneck for others who expect the managers to lead change and ensure that they understand it to provide guidance:

‘… if we can have someone checking on what we do, I think we can even do better.’ (S2, P16, healthcare worker, 44 years, female)

‘… people should ask what we want, I understand this is to assist us and our kids, sometimes it feels like it all about them (nurses), but we are the ones who are to take these treatment, so why we are not involved from beginning (before the decision is made).’ (S4, P3, pregnant woman, 42 years, female)

### Impact of poor communication of changes

Miscommunication of programme changes was cited by most participants. This pertained to healthcare workers who felt there were delays in communicating changes to the programme, which in turn caused delay in its implementation. In addition, patients expressed concerns with poor patient and community involvement, leading to poor participation in their care.

Healthcare workers’ experiences:

‘The information come so late that it takes a while for us to adjust, as you are expected to implement immediately. And you are called in a workshop where there is everyone there … you fail to understand clearly.’ (S2, P11, health care worker, 36 years, female)

Whilst the community caregivers felt that the delay was with their respective PHC facilities, it was clear that healthcare workers too felt they were not adequately involved in the process, as they reported that sometimes the media was their first source of information, such that patients arrived expecting healthcare workers to be aware of developments and provide more information:

‘I first hear from media, but that is not adequate, you expect that as health workers we are to be informed formally … but that delays until you are expected to start implementing the change … this require confidence and be provided with support where you are.’ (S3, P2, healthcare worker, 31 years, female)

Pregnant women as recipients of the programme were pleased with the information they receive from media as it helps to prepare them. However, they feel that with the changes happening so rapidly, they need more time to come to terms and prepare themselves for change:

‘… when I heard in the news I thought it will start the following year, but when I get to clinic I was to be given the drugs … but I was not ready.’ (S2, P10, pregnant woman, 38 years, female)

However, some feel relieved by the change from many drugs to one which seems to be a good change for them:

‘I am happy it is now one pill, so it became easy but I needed time to digest information before starting it.’ (S2, P7, pregnant woman, 30 years, female)

It became clear that not only healthcare workers required time prior to change implementation; the patients also felt strongly about being given enough time to digest this additional information. This is seen as assisting in preparing them for change:

‘… you see … it is not easy, so you still need to be prepared since this change everything to you as well. As much as it helps us, we require more support from our families and nurses and our partners.’ (S2, P4, pregnant woman, 37 years, female)

### Limited communication and engagement with community

Concern was expressed that children still become infected, not because of the failure of the programme or because interventions were not implemented or communicated but because of lack of involvement of communities. As stated by a health care worker:

‘… for this programme to end babies getting disease, the mothers should know we cannot do it without them …. Implementation should be reinforced at different levels, community, us at facilities, the makers of policies and all that are involved.’ (S2, P9, healthcare worker, 36 years, female)

The health care workers, programme coordinators and patients shared their mutual feelings on the role of each person and team approach that lead to the success of the implementation changes. The following quote supports this:

‘I feel the assumption is that facilities alone should make a difference, we need them (communities).’ (S1, P6, healthcare worker, 32 years, female)

The health care worker expresses the need to include not only health facilities but also to actively engage communities.

Pregnant women felt the need to be given enough time to grasp the introduced changes, which for them, makes them part of the process:

‘… us as women I feel we need to be told more, and understand why this is done, the rushing to start is not helping. … this will assist us to contribute.’ (S1, P12, pregnant woman, 22 years, female)

The common theme was that communication of programme changes is generally delayed which has a negative effect on various stakeholders, including consumers.

### Contextual factors embracing innovation in healthcare facilities

Ideas and systems that seemed to be working were in some cases specific to the context of a particular facility. Some of the ideas shared included having a champion for the programme who ensured that each healthcare worker, including the community caregiver, was kept abreast of the changes and checking on performance through audits:

‘… we have a healthcare worker that is allocated to look at all the departments and ensure that every staff member in this hospital is aware and babies are not missed.’ (S2, P9, healthcare worker, 36 years, female)

One site had a category of staff referred to as ‘health system navigators’: personnel employed by a non-governmental organisation acting as a link between the clinic and the patient. They are based at the health facility and phone clients when they do not report to the clinic:

‘… we have health system navigators in this clinic who trace and follow up clients that do not come back.’ (S2, P15, healthcare worker, 29 years, female)

Patients perceive the community caregiver’s role in providing household support as important in enlightening and supporting the patient in her setting:

‘I have a CCG that visit my home once a month, so I discuss with her what I did not understand.’ (S3, P2, pregnant woman, 31 years, female)

Other innovations included use of relevant posters in each healthcare worker’s consulting room that served as a reference for clinicians. Another strategy was having ward-based outreach teams (WBOTS) with a professional nurse and a community caregiver to create demand in the community. As indicated by a healthcare worker:

‘… we have a team here called WBOTS (Ward-Based Outreach Teams) that visits households and they make communities to be aware.’ (S3, P17, healthcare worker, 42 years, female)

### Skill deficits in change management and forward planning

#### Deficits in change management

Whilst innovations were embraced by most managers, as shared by participants, it emerged that healthcare workers felt overwhelmed by frequent changes, such that this impacts on how they manage change; in one instance, lack of space was cited as a problem:

‘… it sometimes takes time for us to start implementing … we start to ask why are we changing now … as it becomes very uncomfortable for us.’ (S1, P18, healthcare worker, 39 years, female)

‘… until we feel we are confident and ready for starting … especially after they (have) taken us through the steps.’ (S3, P1, healthcare worker, 27 years, female)

‘Too much is changing … and no one prepare us for that, and there is not enough space to do all these programmes here.’ (S2, P13, healthcare worker, 33 years, female)

It emerged that planning for change is required by health facilities to ensure that in the event of change, confusion is limited regarding roles.

#### Deficits in forward planning

Although most participants mentioned lack of planning before implementation, it emerged that in some facilities planning played a pivotal role. This was preparing healthcare workers for change implementation. For some clinicians, planning was important because settings differ. Below statement made by health care workers:

‘… provides a plan how it will be implemented here (in the facility) as we are not the same.’ (S3, P2, healthcare worker, 31 years, female)

‘… we hear when we get to clinic and hear the information and we need to make decision the same … this is very difficult because you needed to be prepared.’ (S2, P14, pregnant woman, 23 years, female)

‘… our facility is different and so it becomes important to see how we will do it.’ (S2, P9, healthcare worker, 36 years, female)

Patients felt excluded from decision-making and not given enough time to make decisions:

‘… this is our health, so we need to also play the part.’ (S2, P10, pregnant woman, 38 years, female)

‘… nurses should do this with us. I need to be told what will happen to me, and I need to be given time to decide.’ (S2, P7, pregnant woman, 30 years, female)

Patients still felt uncomfortable with disclosure, especially to their partners:

‘… we need our partners to support us, but sometimes they do not know of our status … because they also don’t tell.’ (S3, P2, pregnant woman, 31 years, female)

The lack of sharing between partners raises concerns in ensuring that patients participate in the interventions for reduction of the transmission rate.

The themes identified from collected data highlighted the experiences and perceptions shared by stakeholders in the effective implementation of changes and in what has worked or has presented challenges for them. It also corroborates the view that disparities exist across health facilities in how the changes are managed which have either a positive or negative impact on the implementation of rapid changes.

### Research on human subjects – Potential benefits and hazards

No risks or benefits were associated with participation in the study.

### Ethical considerations

Permission to conduct the study was obtained from the University of KwaZulu-Natal Biomedical Research Ethics Committee (Reference no. BE112/14) as well as from the KwaZulu-Natal Department of Health Ethics Committee.

## Discussion

In this study, participants described their experiences and perceptions of the implementation of frequent changes to the PMTCT programme, and their realisation that these are needed in an evolving HIV epidemic. Whilst the participants regarded the programme changes as beneficial, there were nonetheless barriers that led to delayed communication of the changes, which affected the way they were adapted for effective implementation. This caused delay in implementation, which could affect PMTCT programme coverage. The participants discussed the gains, the health system challenges and the distress associated with having to deal with high workload and different programmes that demanded their attention. Factors emerged that participants perceived as having worked positively for them over the years in contributing to the improvement of the PMTCT programme.

Experiences of stakeholders in this study corroborate previous findings which showed that changes in and evolution of the PMTCT programme, whilst crucial, did have implications for stakeholders, including both healthcare workers and patients.^16,28^ These implications included amongst others ensuring that all PMTCT programme components are implemented to a high quality in all facilities. Kasenga^28^ reported the increased uptake of the PMTCT programme and antenatal services in Malawi after changes to the PMTCT programme. From the differing settings, both disparities and positive lessons were apparent regarding the frequent changes brought about by newly available knowledge or scientific evidence.

Common gaps appear to be related to communication of the changes and how knowledge is translated to frontline workers. This corroborates a study that looked at how knowledge is translated by looking at knowledge translation models,^29^ most of which suggested that planned knowledge translation for healthcare professionals and consumers is more likely to be successful if the choice of knowledge translation strategy is informed by an assessment of the likely barriers and facilitators. In his theory of innovation, Rogers^30^ pointed out the importance of communication as crucial for implementation.

A study by Haines^31^ highlighted approaches for keeping health professionals’ knowledge up to date. This article drew a similar conclusion that health system constraints or design impede on optimal performance.^32,33,34,35,36^ However, it also appears that focus and engagement with communities are priorities for success in programme implementation.^37^

Leadership support and community involvement, as supported by numerous studies,^10,12,32,35,38^ and particularities of context,^36^ emerged in this study as factors that need to be considered in planning the implementation of changes. A study in South Africa,^36^ which looked beyond policy and focused on experiences to identify health system barriers, highlighted the lack of leadership as contributing to poor implementation.

The stakeholders acknowledged that despite challenges brought about by rapid programme changes, context-specific innovations were nonetheless useful in implementing the changes. Previous studies have noted that PMTCT cascade is monitored through performance of indicators.^12,14^ The article also found that experiences of implementers and consumers had a crucial bearing on effective change implementation. This supports an earlier study conducted in South Africa^36^ which looked at what influences implementation of programmes.

This study suggests a multi-model approach to introducing a change. This includes involvement of communities, support by leadership, effective communication strategies, planning and change management, which should be considered for effective implementation of programme changes.

### Limitations of the study

Having been conducted in four public healthcare facilities in KZN in South Africa, the study may be transferable only to similar resource-limited countries. Further studies could be conducted in other public healthcare facilities in KZN to validate the findings and extend their applicability to similar settings in other provinces and countries. A researcher and research assistant bracketed out through reflexivity, about how their role can shape results interpretation, and avoiding biases. It also ensured that the views and opinions of participants were not influenced in anyway by the researcher and research assistant. Bracketing requires deliberately putting aside one’s own belief about the phenomenon under investigation or what one already knows about the subject prior to and throughout the investigation.^39^ Using standard tools to capture participants’ rich description of experiences also assisted in limiting bias.

### Strengths

The use of multiple sources of evidence ensured triangulation of results and confirmed internal validity. The use of qualitative methodology enriched the study findings as it explored the experiences of various stakeholders in their settings.

## Conclusion

The purpose of this study was to explore the experiences of healthcare workers and patients in the four selected public health facilities during rapid changes to implementation of the PMTCT programme, in KZN, South Africa. The study showed that there can be no one-size-fits-all approach in the implementation of the PMTCT programme changes. Findings suggest that community involvement models, leadership support, effective communication strategies, settings where change is to be affected, planning and change management were amongst the considerations that should be given attention for effective implementation of programme changes for elimination of MTCT.

Innovations exist for effective implementation of the PMTCT programme, which can be useful in meeting set targets for elimination of MTCT; however, accelerated effort to address several factors (health system, healthcare worker and patient-related factors) and specific attention is needed to eliminate contextual disparities for optimal impact on implementation.

## Recommendations

**FIGURE 1:**
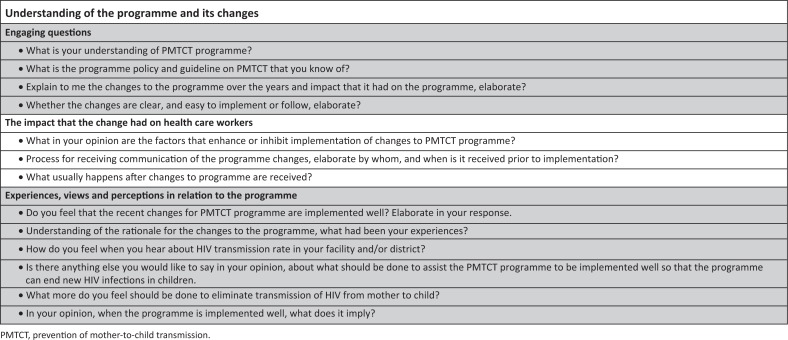
Sample of key questions.

For sub-Saharan countries like South Africa to virtually eliminate MTCT in line with set targets, efforts need to be scaled up and intensified. There is a need to consider various experiences and contexts to manage identified gaps. Below are a few recommendations:

- Planning for change ahead of implementation at all levels including policy makers and considering various contexts.
- Introducing a model that will serve as a guide for implementation of programmes changes, which will include consideration of contexts, community involvement and organisational factors. Provision for a package of high-impact and easy to implement innovations and change ideas that can assist health care workers at implementation level (Figure 1).

## References

[1] World Health Organization (WHO) Global update on HIV treatment 2013: Results, impact and opportunities. Geneva: WHO; 2013.

[2] Joint United Nations Programme on HIV/AIDS (UNAIDS) Global plan towards the elimination of new HIV infections among children by 2015. Geneva: UNAIDS; 2011.

[3] World Health Organization (WHO) Monitoring the building blocks of health systems: A handbook of indicators and their measurement strategies. Geneva: WHO; 2010.

[4] World Health Organization (WHO) Use of antiretroviral drugs for treating pregnant women and preventing HIV infection in infants. Geneva: WHO; 2015. 26180894

[5] Padian NS, McCoy SI, Karim SSA, et al HIV prevention transformed: The new prevention research agenda. Lancet. 2011;378(9787):269–278. https://doi.org/10.1016/S0140-6736(11)60877-5 21763938 10.1016/S0140-6736(11)60877-5 PMC3606928

[6] Joint United Nations Programme on HIV/AIDS (UNAIDS) Global AIDS update 2016. Geneva: UNAIDS; 2016. 12349391

[7] World Health Organization (WHO) Use of antiretroviral drugs for treating pregnant women and preventing HIV infection in infants. Geneva: WHO; 2013. 26180894

[8] Tomlinson M, Doherty T, Jackson D, et al An effectiveness study of an integrated, community-based package for maternal, newborn, child and HIV care in South Africa: Study protocol for a randomized controlled trial. Trials. 2011;12(1):1 https://doi.org/10.1186/1745-6215-12-236 22044553 10.1186/1745-6215-12-236 PMC3248873

[9] Joint United Nations Programme on HIV/AIDS (UNAIDS) Fast-track: Ending the AIDS epidemic by 2030. Geneva: UNAIDS; 2014.

[10] Sidibé M, Loures L, Samb B The UNAIDS 90–90–90 target: A clear choice for ending AIDS and for sustainable health and development. J Int AIDS Soc. 2016;19:1–2. https://doi.org/10.7448/IAS.19.1.21133 10.7448/IAS.19.1.21133 PMC4947868 27424601

[11] Department of Health (DOH) South Africa’s national strategic plan for HIV, TB and STIs 2017–2022 [homepage on the Internet]. 2017 [cited 2016 March 20]. Available from: www.sanac.org.za

[12] Bhardwaj S, Carter B, Aarons GA, Chi BH Implementation research for the prevention of mother-to-child HIV transmission in sub-Saharan Africa: Existing evidence, current gaps, and new opportunities. Curr HIV/AIDS Rep. 2015;12(2):246–255. https://doi.org/10.1007/s11904-015-0260-1 25877252 10.1007/s11904-015-0260-1 PMC4430362

[13] Barron P Re-engineering primary health care in South Africa: Discussion document. Department of Health, Presentation; 2010 November 02; held at Umhlanga Rocks, South Africa 2010.

[14] Barker P, Barron P, Bhardwaj S, Pillay Y The role of quality improvement in achieving effective large-scale prevention of mother-to-child transmission of HIV in South Africa. AIDS. 2015;29:S137–S143. https://doi.org/10.1097/QAD.0000000000000718 26102624 10.1097/QAD.0000000000000718

[15] Barron P, Pilly Y, Doherty T, et al Eliminating mother-to-child transmission in South Africa. *Bulletin of the Health Orginization*. 2013;91(1):70–74. https://doi.org/10.2471/BLT.12.106807 10.2471/BLT.12.106807 PMC3537246 23397353

[16] Barron P Eliminatig mother to child transmission of HIV in South Africa. WHO Bull. 2013;91:70–74. 10.2471/BLT.12.106807 PMC3537246 23397353

[17] National Department of Health (NDOH) National consolidated guidelines for the prevention of mother-to-child transmission of HIV (PMTCT) and the management of HIV in children, adolescents and adults. Pretoria: NDOH; 2015.

[18] National Department of Health (NDOH) National consolidated guidelines for the prevention of mother-to-child transmission of HIV (PMTCT) and the management of HIV in children, adolescents and adults. Pretoria: NDOH; 2013.

[19] Denzin NK, Lincoln Y Qualitative research Thousand Oaks, CA: Sage; 2000.

[20] Statistics South Africa (StatsSA) Mid-year population estimates. Pretoria: Statistics South Africa; 2016.

[21] KwaZulu-Natal Department of Health (KZNDOH) District Health Information System (DHIS). 2015 (Unpublished data).

[22] Glaser BG, Strauss AL The discovery of grounded theory: Strategies for qualitative theory. New Brunswick: Aldine Transaction; 1967.

[23] Cohen L, Manion L, Morrison K Research methods in education 5th ed. RoutledgeFalmer, London 2002.

[24] Beck CT, Polit DF Essential of nursing reserach: Appraising evidence for nursing practic. Philadelphia: Wolters Kluwer, Lippincott Williams & Wilkins; 2010.

[25] Creswell JW, Miller DL Determining validity in qualitative inquiry. Theory Practice. 2000 Aug 1;39(3):124–130.

[26] Polit DF, Hungler BP The analysis of qualitative data In: Polit DF, Hungler BP, editors Nursing research: Principles and methods. Philadelphia: Wolters Kluwer, Lippincott Williams & Wilkins; 1999; p. 573–590.

[27] Miles M, Huberman A Qualitative data analysis. Thousand Oaks, CA: Sage; 1994.

[28] Kasenga F, Byass P, Emmelin M, Hurtig A-K The implications of policy changes on the uptake of a PMTCT programme in rural Malawi: First three years of experience. Glob Health Action. 2009;2 https://doi.org/10.3402/gha.v2i0.1883 10.3402/gha.v2i0.1883 PMC2779935 20027274

[29] Grimshaw JM, Eccles MP, Lavis JN, Hill SJ, Squires JE Knowledge translation of research findings. Implement Sci. 2012;7:1 https://doi.org/10.1186/1748-5908-7-50 10.1186/1748-5908-7-50 PMC3462671 22651257

[30] Rogers EM Diffusion of innovations. 4th ed. New York: Free Press; 1995.

[31] Chabikuli O, Gwarzo U, Olufunso A, et al Closing the prevention of mother-to-child transmission gap in Nigeria: An evaluation of service improvement intervention in Nigeria. S Afr Fam Pract. 2013;55:96–102. https://doi.org/10.1080/20786204.2013.10874310

[32] Haines A Bridging the implementation gap between knowledge and action for health. Bull World Health Organ. 2004;82:724–731. 15643791 PMC2623035

[33] Doherty T, Chopra M, Nsibande D, Mngoma D Improving the coverage of the PMTCT programme through a participatory quality improvement intervention in South Africa. BMC Pub Health. 2009;9:1 https://doi.org/10.1186/1471-2458-9-406 19891775 10.1186/1471-2458-9-406 PMC2777166

[34] Doherty TM, Mccoy D, Donohue S Health system constraints to optimal coverage of the prevention of mother-to-child HIV transmission programme in South Africa: Lessons from the implementation of the national pilot programme. Afr Health Sci. 2005;5:213–218. 16245991 10.5555/afhs.2005.5.3.213 PMC1831931

[35] Sprague C, Chersich MF, Black V Health system weaknesses constrain access to PMTCT and maternal HIV services in South Africa: A qualitative enquiry. AIDS Res Ther. 2011;8(10). https://doi.org/10.1186/1742-6405-8-10 10.1186/1742-6405-8-10 PMC3058008 21371301

[36] Byamugisha R, Tumwine JK, Semiyaga N, Tylleskar T Determinants of male involvement in the prevention of mother-to-child transmission of HIV programme in Eastern Uganda: A cross-sectional survey. Reprod Health. 2010;7(12). https://doi.org/10.1186/1742-4755-7-12 10.1186/1742-4755-7-12 PMC2913932 20573250

[37] Uwimana J, Jackson D, Hausler H, Zarowsky C Health system barriers to implementation of collaborative TB and HIV activities including prevention of mother-to-child transmission in South Africa. Trop Med Int Health. 2012;17:658–665. https://doi.org/10.1111/j.1365-3156.2012.02956.x 22394016 10.1111/j.1365-3156.2012.02956.x

[38] Aizire J, Fowler G, Coovadia H Operational issues and barriers to implementation of prevention of mother-to-child transmission of HIV (PMTCT) interventions in sub-Saharan Africa. Curr HIV Res. 2013;11:144–159. https://doi.org/10.2174/1570162X11311020007 23432490 10.2174/1570162x11311020007

[39] Mofesson LM, Siberry GK, Watts DH, et al A multi-disciplinary approach to implementation science: The NIH-PEPFAR PMTCT implementation science alliance. J Acquir Immune Defic Syndr. 2014;67:S163–S167. 25310124 10.1097/QAI.0000000000000323

